# Single mutations in the ε subunit from thermophilic *Bacillus* PS3 generate a high binding affinity site for ATP

**DOI:** 10.7717/peerj.5505

**Published:** 2018-09-05

**Authors:** Alexander Krah, Peter J. Bond

**Affiliations:** 1 School of Computational Sciences, Korea Institute for Advanced Study, Seoul, Republic of Korea; 2 Department of Biophysics, Graduate School of Science, Kyoto University, Kyoto, Japan; 3 Bioinformatics Institute, Agency for Science, Technology and Research (A*STAR), Singapore, Singapore; 4 Department of Biological Sciences, National University of Singapore, Singapore, Singapore

**Keywords:** ε subunit, ATP synthase, ATP binding, MD simulations, Binding affinity

## Abstract

The ε subunit from ATP synthases acts as an ATP sensor in the bacterial cell to prevent ATP hydrolysis and thus the waste of ATP under conditions of low ATP concentration. However, the ATP binding affinities from various bacterial organisms differ markedly, over several orders of magnitude. For example, the ATP synthases from thermophilic *Bacillus* PS3 and *Escherichia coli* exhibit affinities of 4 µM and 22 mM, respectively. The recently reported R103A/R115A double mutant of *Bacillus* PS3 ATP synthase demonstrated an increased binding affinity by two orders of magnitude with respect to the wild type. Here, we used atomic-resolution molecular dynamics simulations to determine the role of the R103A and R115A single mutations. These lead us to predict that both single mutations also cause an increased ATP binding affinity. Evolutionary analysis reveals R103 and R115 substitutions in the ε subunit from other bacillic organisms, leading us to predict they likely have a higher ATP binding affinity than previously expected.

## Introduction

ATP synthases are enzymes which couple rotary ATP synthesis to an electrochemical ion gradient across a lipid bilayer (Diez et al., 2004). They are also able to act reversibly, driving the electrochemical gradient by hydrolysing ATP, resulting from rotation in the opposite direction (Noji et al., 1997). Bacteria and mitochondria have developed both, common and unique mechanisms of ATP synthase regulatory inhibition to prevent wasteful ATP hydrolysis. A common mechanism among species is based on Mg^2+^-ADP induced inhibition (Hirono-Hara et al., 2001); additional regulation involves the protein IF_1_ in mitochondria or the ε subunit in bacteria, respectively, as summarized in a recent review (Krah, 2015). In addition, ATP synthases from α-proteobacteria are regulated by subunit *ζ* (Morales-Ríos et al., 2010; Zarco-Zavala et al., 2014) and others have been proposed to not hydrolyse ATP (McMillan et al., 2016).

**Figure 1:**
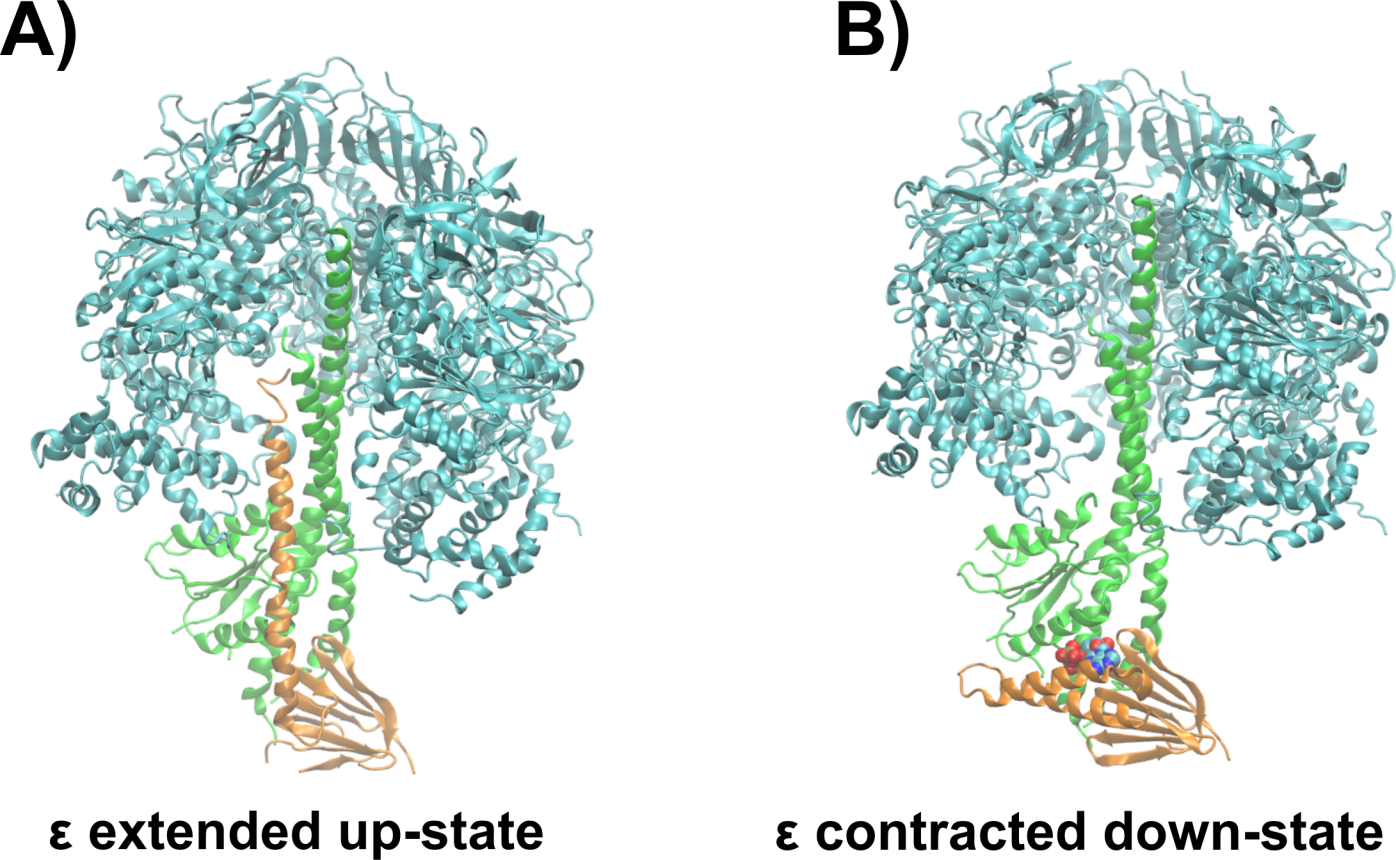
Structural basis of ATPase inhibition and non-inhibition by subunit ε. Representation of the extended up-state and the contracted down-state of the ε subunit in the *F*_1_ domain from thermophilic *Bacillus* PS3 is shown in (A) and (B), respectively. The ε subunit and the central stalk *γ* are shown in orange and green, respectively. One β subunit of the hexameric assembly is omitted for clarity.

The ε subunit from bacterial ATP synthases is a small protein consisting of two distinct domains, a rigid N-terminal β-sandwich domain and a flexible α-helical C-terminal domain (Wilkens et al., 1995; Uhlin, Cox & Guss, 1997; Wilkens & Capaldi, 1998; Yagi et al., 2007; Yagi et al., 2010). In certain bacterial organisms, the C-terminal domain acts as an ATP sensor, which is selective in the wild type protein from thermophilic *Bacillus* PS3 over other nucleotides, as ADP, GTP, CTP, and UTP do not bind to the ε subunit according to gel filtration experiments (Kato-Yamada & Yoshida, 2003). The C-terminal domain undergoes a large conformational change from the non-inhibitory down- to the ATPase inhibitory up-state, when ATP unbinds upon passing a crucial concentration threshold (Iino et al., 2005; Feniouk et al., 2010). In the ATP-bound conformation (down-state), the C-terminal domain adopts a contracted conformation, with both α-helices in a hairpin-like fold, and residues from both α-helices interact with ATP (Yagi et al., 2007). Molecular dynamics (MD) simulations recently revealed that, in addition, a Mg^2+^ ion unresolved in the crystal structure (Yagi et al., 2007) is also bound to the ATP molecule (Krah & Takada, 2016). After ATP release, the ε subunit first adopts a half-extended conformation (Rodgers & Wilce, 2000) and is then captured in the fully extended conformation (up-state) to interact with the catalytic subunits of the enzyme (Cingolani & Duncan, 2011; Shirakihara et al., 2015; Sobti et al., 2016). The ATP bound down-state of the ε subunit has been proposed to allow coupling between ATPase activity and proton translocation (Kadoya et al., 2011). A detailed discussion of the regulation of bacterial ATP synthases by the ε subunit can be found elsewhere (Krah, Zarco-Zavala & McMillan, 2018). Figure 1 shows the whole ATP synthase from thermophilic *Bacilus* PS3, highliting the position of the ε subunit.

The ATP binding affinities from different organisms range from the µM to the mM range, as measured for the isolated ε subunit from thermophilic *Bacillus* PS3 (*K*_*d*_(ATP) = 4.3 µM) (Kato, Yoshida & Kato-Yamada, 2007), *Bacillus subtilis* (*K*_*d*_(ATP) = 2.3 mM) (Kato-Yamada, 2005) or *Escherichia coli* (*K*_*d*_(ATP) = 22 mM) (Yagi et al., 2007), indicating different physiological functions of the ε subunit from different organisms. The average ATP concentration in cells is 3.2 mM (Traut, 1994). Under these conditions, in accordance with their *K*_*d*_(ATP) values, the ε subunit from thermophilic *Bacillus* PS3 and *B. subtilis* would adopt an up- and down- state, respectively. The ATP concentration in *E. coli* cells is on average 1.5 mM (Yaginuma et al., 2014), and 9.6 mM in the glucose fed state (Bennett et al., 2009)—thus, the ε subunit from *E. coli* would not appreciably bind ATP under physiological conditions and would mainly be expected to reside in the up-state. In addition, recent biophysical experiments revealed that the R103A/R115A double-mutant of the ε subunit from thermophilic *Bacillus* PS3 binds ATP with a two orders of magnitude increase in affinity (52 nM) compared to the wild type (Kato-Yamada, 2016), which is caused by an enhanced hydrogen bonding network and a loss of repulsive contacts of the Mg^2+^ ion and basic protein residues with other positively charged residues at the binding site (Krah, Kato-Yamada & Takada, 2017); the position of the Mg^2+^ ion was shown to be important for the enthalpic contributions of ATP binding to the protein in the same study. Understanding the molecular effect of individual non-ligand binding residues upon the binding affinity may facilitate the prediction of affinities of the ε subunit from different organisms based on sequence comparison. In addition, an improved knowledge of non-binding residues that nevertheless influence the ligand binding affinity may help to fine-tune genetically encoded ATP sensors, based on subunit ε (Imamura et al., 2009; Yaginuma et al., 2014), which have been shown to be applicable to various medical and biological problems, such as measurement of intramitochondrial ATP concentration during hypoxia (Kioka et al., 2014), the mechanism of anaesthetics on mitochondrial ATP synthesis (Kishikawa et al., 2018) or the quantification of ATP levels of living cells infected by a virus (Ando et al., 2012).

In this study, we used atomic-resolution, explicitly solvated MD simulations to predict the structural and energetic basis for ATP binding in the individual R103A and R115A mutants, comparing the resultant properties with the wild type (Krah & Takada, 2016) and the R103A/R115A double mutant (Krah, Kato-Yamada & Takada, 2017). Our main findings were that both single mutants bind ATP via a more pronounced hydrogen-bonding network compared to the wild type, but similar to the R103A/R115A double mutant, predicting a ligand binding affinity in the nM range for both single mutants. Based on a sequence alignment, we predict that the ε subunit from other bacillic organisms binds ATP in the nM range. Although R115 and R103 seem to be highly conserved among bacillic organisms, we found these residues to be substituted by other amino acids on occasion, leading us to hypothesize an increased binding affinity for some of them.

## Material and Methods

### Conventional molecular dynamics simulations

**Figure 2:**
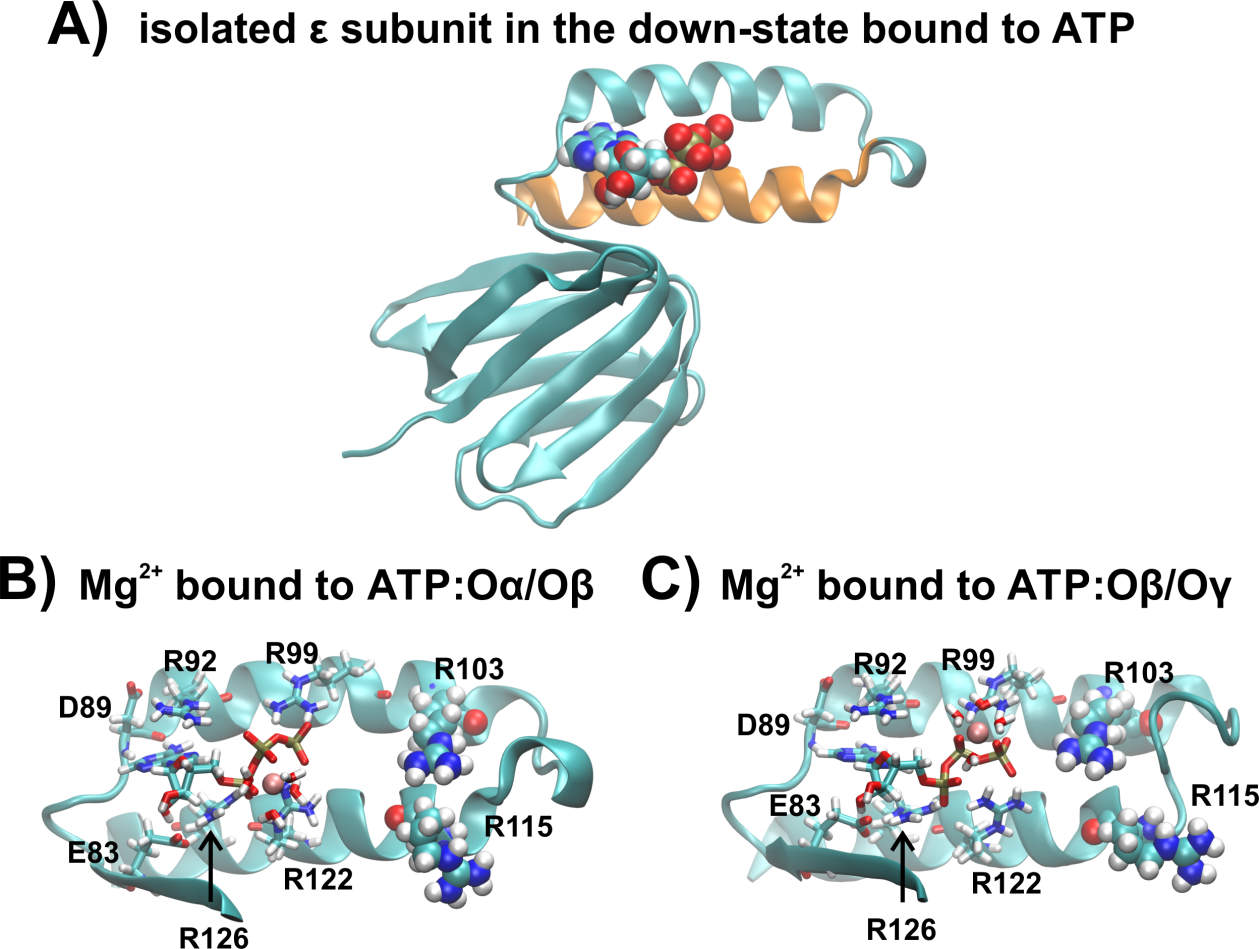
The ε subunit in the down-state (A) Graphical representation of the wild type ε subunit from thermophilic *Bacillus* PS3 in the down state. The second α-helical domain is shown in orange. (B) and (C) show a zoom into the ATP binding site, when ATP is bound to ATP:O α/Oβ or ATP:O β/O*γ*, respectively. Binding site residues are shown in licorice, while R103 and R115 are shown in VdW spheres.

Conventional MD simulations were carried out for the R103A and R115A single mutants of the ε subunit from thermophilic *Bacillus* PS3 for three different conditions: (i) We obtained the starting structures from our previous wild-type simulation (Krah & Takada, 2016), introducing the single mutations R103A and R115A. Four Mg^2+^ ions were freely distributed in bulk solution and no ion was initially bound to ATP, and this was simulated for 150 ns in triplicate. Subsequently, the final snapshots from these simulations were extracted, and a single Mg^2+^ ion was modelled in the first sphere of coordination to (ii) ATP:Oα/Oβ or (iii) ATP:Oβ/O*γ*, with the remaining three Mg^2+^ ions remaining freely distributed in the system. These two latter systems were simulated for 100 ns, each in triplicate. The isolated wild type ε subunit and a zoomed image of the ATP binding site, clarifying the coordination of Mg^2+^ to ATP:Oα/Oβ and ATP:Oβ∕O*γ*, is shown in Fig. 2.

The simulations were carried out with the simulation suite GROMACS (version 4.6.5) (Pronk et al., 2013), applying the AMBER-ILDN force-field parameters (Cornell et al., 1995; Wang, Cieplak & Kollman, 2000; Meagher, Redman & Carlson, 2003; Hornak et al., 2006; Lindorff-Larsen et al., 2010) as implemented in GROMACS (Sorin & Pande, 2005). Counter ions were added to neutralize the system. Protonation states of ionisable residues were set as in the wild type simulations (Krah & Takada, 2016). The TIP3P water model (Jorgensen et al., 1983) was used. Temperature and pressure were kept constant at 300K and 1 bar, respectively, using the velocity-rescale thermostat (Bussi, Donadio & Parrinello, 2007) and Parinello-Rahman barostat (Parrinello & Rahman, 1981). To calculate electrostatic interactions, the Particle Mesh Ewald method was used, applying a real space cut-off of 12 Å. An integration time step of 2 fs was applied for all simulations, constraining bonds containing hydrogen atoms using the LINCS algorithm (Hess et al., 1997).

### Analysis of equilibrium simulations

To rationalize the experimental data, we analysed the protein hydrogen bond network with bound ATP. Additionally, the protein-protein hydrogen bond network, between the second α-helical C-terminal domain (residues 112 to residue 133) and the remainder of the protein, was examined. For measuring a hydrogen bond, a cut-off distance of 2.7 Å from the hydrogen (donor) to the acceptor was chosen. A cut-off of 30° for the angle between donor, hydrogen, and acceptor was applied. The energy contribution of the hydrogen-bond network was estimated using the method as proposed by Espinosa, Molins & Lecomte (1998), which estimates the hydrogen-bond energy by taking into account the distance between hydrogen bond donor and acceptor atoms. The first 10 ns of the trajectory were considered as equilibration and discarded prior to analysis. In addition, we defined repulsive electrostatic contacts, i.e., positively charged residues located in the binding site, including the Mg^2+^ ion, using a maximum cut-off distance of 4.5 Å. These contacts destabilize the binding site via interactions between positively charged residues (R92, R99, R103, R115, R122 and R126) with one another and/or the Mg^2+^ ion. Standard deviations were calculated using the averages of all three runs.

### Free energy calculations

To clarify if the Mg^2+^ ion is bound in a first (Mg^2+^ bound to ATP:Oα/Oβ or ATP:Oβ/O*γ*) or second coordination (Mg^2+^ bound to ATP via bridging water molecules) sphere, we calculated the solvation free energy of the ion in these different states, using a representative structure obtained from conventional MD simulations as an input, after removing all other Mg^2+^ ions which were not bound to ATP. To calculate the solvation free energy, the thermodynamic integration (TI) method was used, with a total of 85 windows per calculation, as described previously (Krah & Takada, 2016). Van der Waals and electrostatic interactions were removed linearly. To keep the ion in the intended position, a restraint potential with force constant of 1.5 kcal mol^−1^ Å^−2^ was applied. Forward and backward TI calculations were carried out for 500 ps per window, considering the first 100 ps as equilibration time. Analysis of the results was carried out with the g_bar GROMACS module.

## Results

### Simulations of the protein-ligand complex—Mg^2+^ freely distributed in solution

The ATP binding affinities for the wild type (Kato, Yoshida & Kato-Yamada, 2007) and the R103A/R115A double mutant (Kato-Yamada, 2016) for the ε subunit from thermophilic *Bacillus* PS3 have been measured recently, revealing a difference of two orders of magnitude in favour of the mutant (4.3 µM versus 52 nM, respectively). We recently showed that this remarkable increase in ligand binding affinity in the R103A/R115A double mutant (Kato-Yamada, 2016) is caused by an extended hydrogen-bond network and decreased repulsive contacts of positively charged residues, including the Mg^2+^ ion, between one another within the ATP binding site (Krah, Kato-Yamada & Takada, 2017). To examine the influence on structural and biophysical properties of both single mutations, namely R103A and R115A, on the ε subunit from thermophilic *Bacillus* PS3, we conducted MD simulations of these mutants. First, we simulated a system in which Mg^2+^ is freely distributed in solution but not initially bound to ATP. During the simulations, we observed for both mutants the spontaneous binding of the Mg^2+^ ion into a second sphere coordination in the vicinity of the ATP molecule, as also observed previously for the wild type ε subunit from thermophilic *Bacillus* PS3 and *Bacillus subtilis* (Krah & Takada, 2015; Krah & Takada, 2016). The results are shown in Fig. S1.

### Simulations of the protein-ligand complex—Mg^2+^ bound to ATP in a first sphere coordination

**Figure 3:**
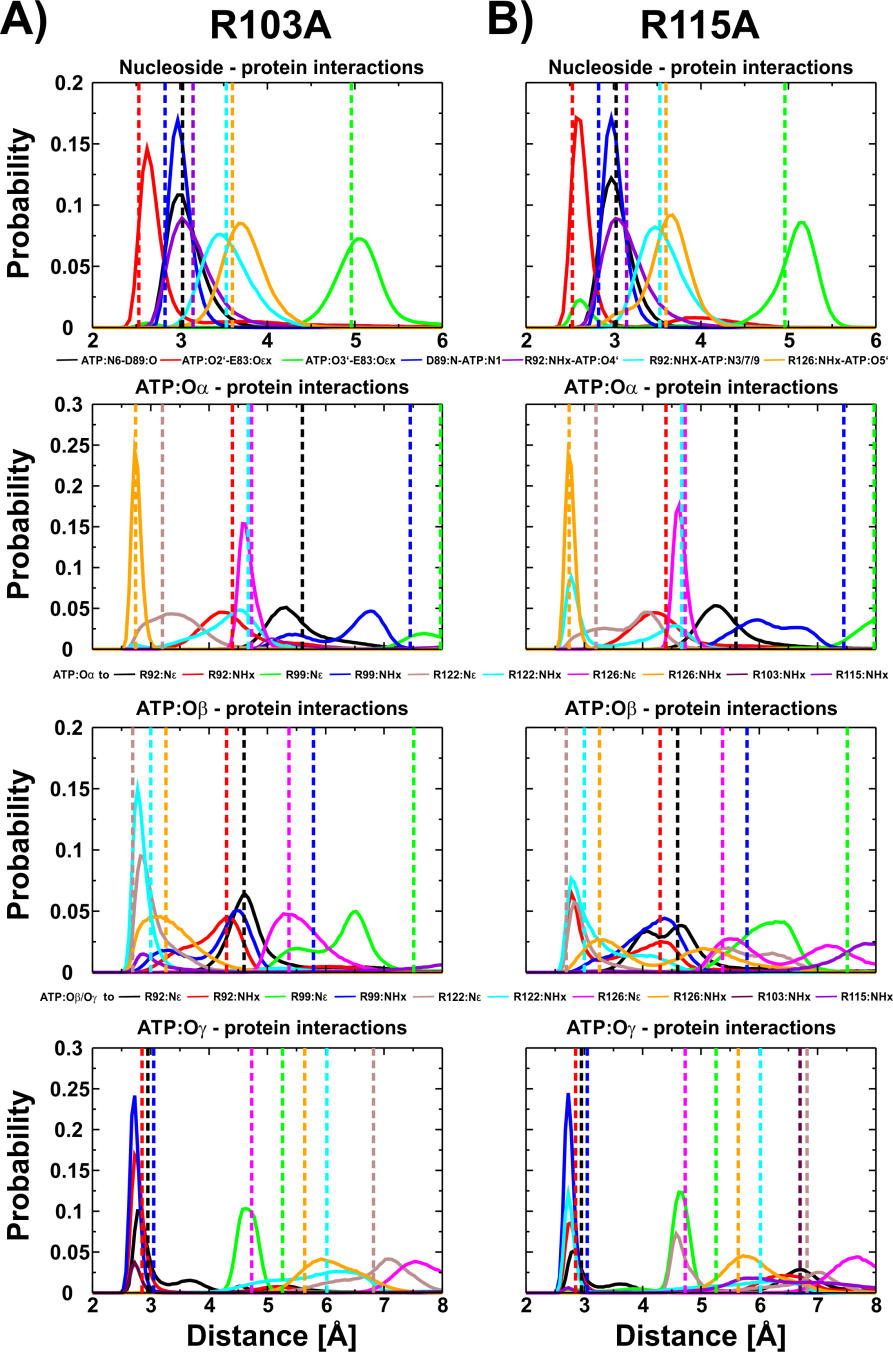
ATP interactions during simulation. Interactions of the ε subunit R103A and R115A mutants from thermophilic *Bacillus* PS3 with ATP during the simulations when Mg^2+^ is coordinated by ATP:Oα/Oβ are shown in (A) and (B), respectively. The corresponding figure for the ATP:Oβ/O*γ* ion coordination state can be found in Fig. S3. Dotted lines correspond to the distances observed in the crystal structure of the wild type protein (PDB-ID: 2E5Y) (Yagi et al., 2007).

We have shown for the wild type (Krah & Takada, 2016) and the R103A/R115A (Krah, Kato-Yamada & Takada, 2017) double mutant of the ε subunit from thermophilic *Bacillus* PS3 that a Mg^2+^ ion is bound to ATP:Oα/Oβ, as later also confirmed by X-ray crystallography for the ε subunit from *Caldalkalibacillus thermarum* (Ferguson et al., 2016), which shares the same ATP binding residues (E83, R92, R99, R123 and R127) (Yagi et al., 2007; Krah & Takada, 2016). In the wild type protein and the R103A/R115A mutant, the stability and energetics of ATP binding to the ε subunit is dependent upon the position of the Mg^2+^ ion (Krah & Takada, 2016; Krah, Kato-Yamada & Takada, 2017). Taking these previous findings into account, we studied whether a Mg^2+^ ion is bound to ATP in both single mutants (R103A and R115A). We thus carried out conventional MD simulations for both mutants with the Mg^2+^ ion bound to ATP:Oα/Oβ or ATP:Oβ/O*γ*. Most interactions of the protein with ATP were stable in both mutants. The RMSD of the wild type (Krah & Takada, 2016), the double mutant (Krah, Kato-Yamada & Takada, 2017) and the two single mutants is comparable as shown in Table S1. Compared to the wild type (Krah & Takada, 2016), we observed stabilized E83:Oε *x* − *ATP*:*O*2′ (Fig. S2) interactions for both mutants, in which the Mg^2+^ ion is coordinated by ATP:Oα/Oβ (Fig. 3), while this interaction was significantly disturbed when the Mg^2+^ ion is bound to ATP:Oβ/O*γ* (Figs. S2 and S3). All other nucleoside interactions remained stable in both systems (Fig. 3). In addition, we observed an increased stability of the protein-ATP:O*γ* interactions for both mutants if the Mg^2+^ ion is coordinated by ATP:Oα/Oβ rather than ATP:O β/O*γ*, and additional favourable protein-ATP:O β interactions for the R103A mutant depending on whether the Mg^2+^ ion is bound to ATP:Oα/Oβ or ATP:Oβ/O*γ*, respectively (Fig. 3, Figs. S2 and S3). These results indicate that the Mg^2+^ ion is most likely coordinated by ATP:Oα/Oβ in both single mutants, as previously predicted by MD simulations for the wild type (Krah & Takada, 2016) and the R103A/R115A mutant (Krah, Kato-Yamada & Takada, 2017) protein from thermophilic *Bacillus* PS3, as also shown by X-ray crystallography of the ε subunit from *C. thermarum* (Ferguson et al., 2016). It should be noted that one simulation replica of the R115A mutant (Mg^2+^ bound to ATP:Oα/Oβ) was excluded from analysis, as the simulation was assessed to be insufficiently converged. This was based on the observation that R103 (bound to ATP:O*γ*) prevented R122 from binding to ATP:O*γ*, which is likely to lower the binding affinity drastically, as indicated by the experimental alanine mutant (R122A) (Kato, Yoshida & Kato-Yamada, 2007); R122 coordinates the ligand in the wild-type (Yagi et al., 2007; Krah & Takada, 2016). Interactions of R103:NHx and R122:NHx to ATP of this single run are shown in Fig. S4.

### Is the Mg^2+^ ion bound to ATP:Oα/Oβ or ATP:Oβ/O*γ* in the protein-ligand complex?

**Figure 4:**
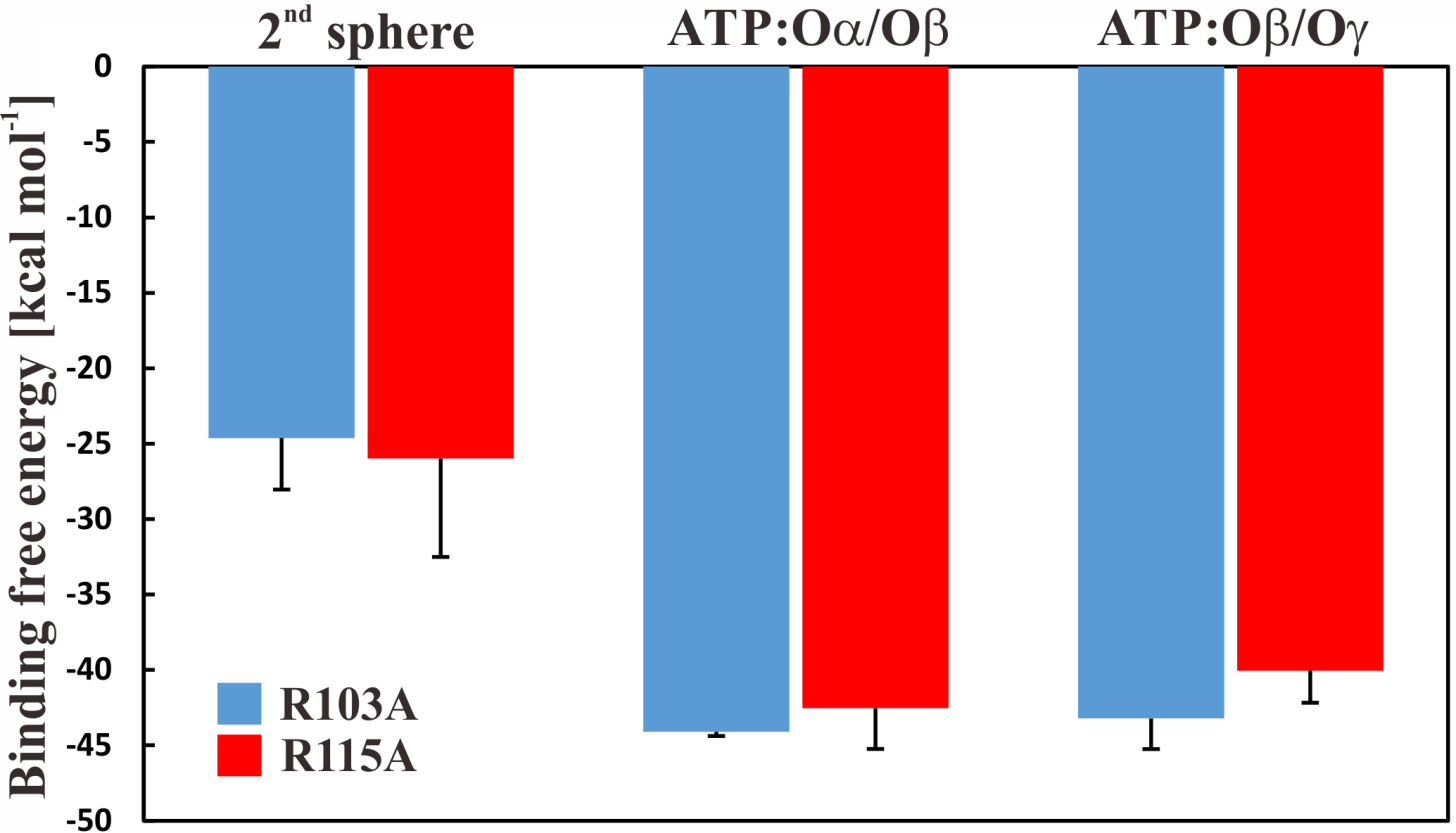
Binding free energy of Mg^2+^ to ATP. Binding free energy for the Mg^2+^ ion in different states for the R103A and R115A single mutant of the ε subunit from thermophilic *Bacillus* PS3. The values correspond to the difference in solvation free energy of a Mg^2+^ ion in the bulk versus at the binding site. The error bars represent the standard deviation of the forward and backward calculations.

To further study if—and by which phosphate atoms—the ion is coordinated with ATP, we first applied free energy calculations. We carried out thermodynamic integration calculations in the forward and backward directions, which suggested that the ion is more favourably coordinated in a first sphere of coordination (the Mg^2+^ ion is bound to ATP and four water molecules), by ∼20 kcal mol^−1^ with a slight preference for the Mg^2+^ ion to be coordinated by the ATP:Oα/Oβ atoms. The results are shown in Fig. 4; we further conclude that the free energy calculations are converged because calculations in both directions show similar results (Fig. S5). It should be reiterated that the parametrization of divalent ions is typically problematic in classical force-fields, as discussed elsewhere (Li et al., 2015). However, the results are similar and within the error bars. To clarify the position of the ion, we calculated the enthalpic contribution (Espinosa, Molins & Lecomte, 1998) for both states in both mutant systems; the entropic contributions are assumed to be similar in both ion coordination states. We observe that the enthalpic contribution is remarkably favourable for both single mutants, if Mg^2+^ is bound to ATP:Oα/Oβ, rather than to ATP:Oβ/O*γ* (Table 1).

**Table 1:** Protein-ligand interactions based on MD simulations. (a) The mean number of hydrogen bonds between the protein and ATP, internal protein–protein hydrogen bonds of the C-terminal second -helix to the rest of the protein, and repulsive contacts in the subunit from the wild type, the R103A/R115A double-mutant, and the R103A and R115 single-mutants. (b) Energetic analysis of the hydrogen bonding network with ATP and the flexible α-helices. Oa/Oβ and Oβ/Oγ denote the ATP oxygen atoms to which the Mg^2+^ ion is coordinated during the simulation. The results from the wild type and R103A/R115A double mutant have been adapted from Krah, Kato-Yamada & Takada (2017).

	Wild type		R103A/R115A	
	Oα/Oβ	Oβ/O*γ*	Oα/Oβ	Oβ/O*γ*
(a)				
h-bonds (protein-ATP)	9.38 ± 0.48	9.45 ± 0.09	10.4 ± 0.60	9.74 ± 0.47
h-bonds (2nd α-helix)	5.27 ± 0.20	4.93 ± 0.69	4.01 ± 0.18	4.05 ± 0.26
repulsive contacts	1.96 ± 0.51	1.58 ± 0.20	1.08 ± 0.51	1.54 ± 0.06

	R103A		R115A	
	O α/Oβ	O β/O*γ*	O α/Oβ	O β/O*γ*
h-bonds (protein-ATP)	10.78 ± 0.25	9.94 ± 0.95	10.83 ± 0.01	9.00 ± 0.46
h-bonds (2nd α-helix)	5.14 ± 0.70	5.14 ± 1.55	4.33 ± 0.11	4.44 ± 0.44
repulsive contacts	1.83 ± 0.58	1.88 ± 0.65	1.53 ± 0.44	1.46 ± 0.45

	Wild type (kcal/mol)		R103A/R115A (kcal/mol)	
	Oα/Oβ	Oβ/O*γ*	Oα/Oβ	Oβ/O*γ*
(b)				
E_HB_ (h-bonds (protein-ATP))	−64.6 ± 2.4	−65.2 ± 0.1	−73.4 ± 2.1	−67.8 ± 2.9
E_HB_ (h-bonds (2nd α-helix))	−28.6 ± 0.7	−27.0 ± 2.3	−26.4 ± 1.5	−27.4 ± 2.3
E_HB_	−93.2 ± 0.31	−92.2 ± 0.42	−99.8 ± 1.8	−95.2 ± 2.6

	R103A (kcal/mol)		R115A (kcal/mol)	
	O α/Oβ	O β/O*γ*	O α/Oβ	O β/O*γ*
E_HB_ (h-bonds (protein-ATP))	−73.4 ± 2.6	−68.9 ± 6.4	−76.0 ± 1.7	−60.5 ± 4.3
E_HB_ (h-bonds (2nd α-helix))	−29.3 ± 2.1	−29.8 ± 8.3	−27.3 ± 0.7	−27.6 ± 3.6
E_HB_	−102.7 ± 2.3	−98.6 ± 7.4	−103.3 ± 2.7	−88.1 ± 4.0

To derive a detailed energetic picture of ATP binding to subunit ε, we analysed the hydrogen bond energetics, which are dependent upon the distance of the hydrogen bond donor and acceptor atoms, as previously introduced by Espinosa et al. (Espinosa, Molins & Lecomte, 1998). We used this method to estimate the energy of protein-ATP interactions and the energy of interaction between the second helix, which undergoes a large conformational change upon ATP unbinding, with the remainder of the protein. Our results show that for both mutants the Mg^2+^ coordination to ATP:Oα/Oβ is energetically more favourable than if the Mg^2+^ binds to ATP:Oβ/O*γ*, which is in agreement with the free energy calculations for both single mutants. The results of the energetic analysis, showing the data for the hydrogen binding network and the consequent enthalpic contribution of the single mutants, the wild type and the double mutant (Krah, Kato-Yamada & Takada, 2017), are summarized in Table 1.

### Predicting the binding affinity of the R103A and R115A mutants

Considering the energetic analysis, we predict that both single mutants (R103A and R115A) of the ε subunit from thermophilic *Bacillus* PS3 should have increased binding affinities to the ligand with respect to wild-type: the hydrogen bonding energy (E_HB_) of the mutant proteins interacting with the ligand becomes significantly more favourable. The more favourable interaction energy is caused by an increased number of hydrogen bonds (Table 1) and slightly adjusted interaction distances of binding site residues with the ligand (Fig. 3, Krah & Takada, 2016; Krah, Kato-Yamada & Takada, 2017). The contribution of the flexible C-terminal α-helical domain is in each case similar, and the repulsive inter-atomic contacts between positively charged residues (including the Mg^2+^ ion) are reduced (Table 1). As the R103A/R115A double mutant binds ATP with an affinity of 52 nM (Kato-Yamada, 2016), we hypothesize that both single mutants bind in the nM range. It should be mentioned that previous gel-filtration experiments also showed ATP binding to the R115A mutant (Kato, Yoshida & Kato-Yamada, 2007); however, the binding affinity of the R115A mutant was not investigated in this study.

### The binding sites of the R103A and R115A mutants of the ε subunit from thermophilic *Bacillus* PS3

**Figure 5:**
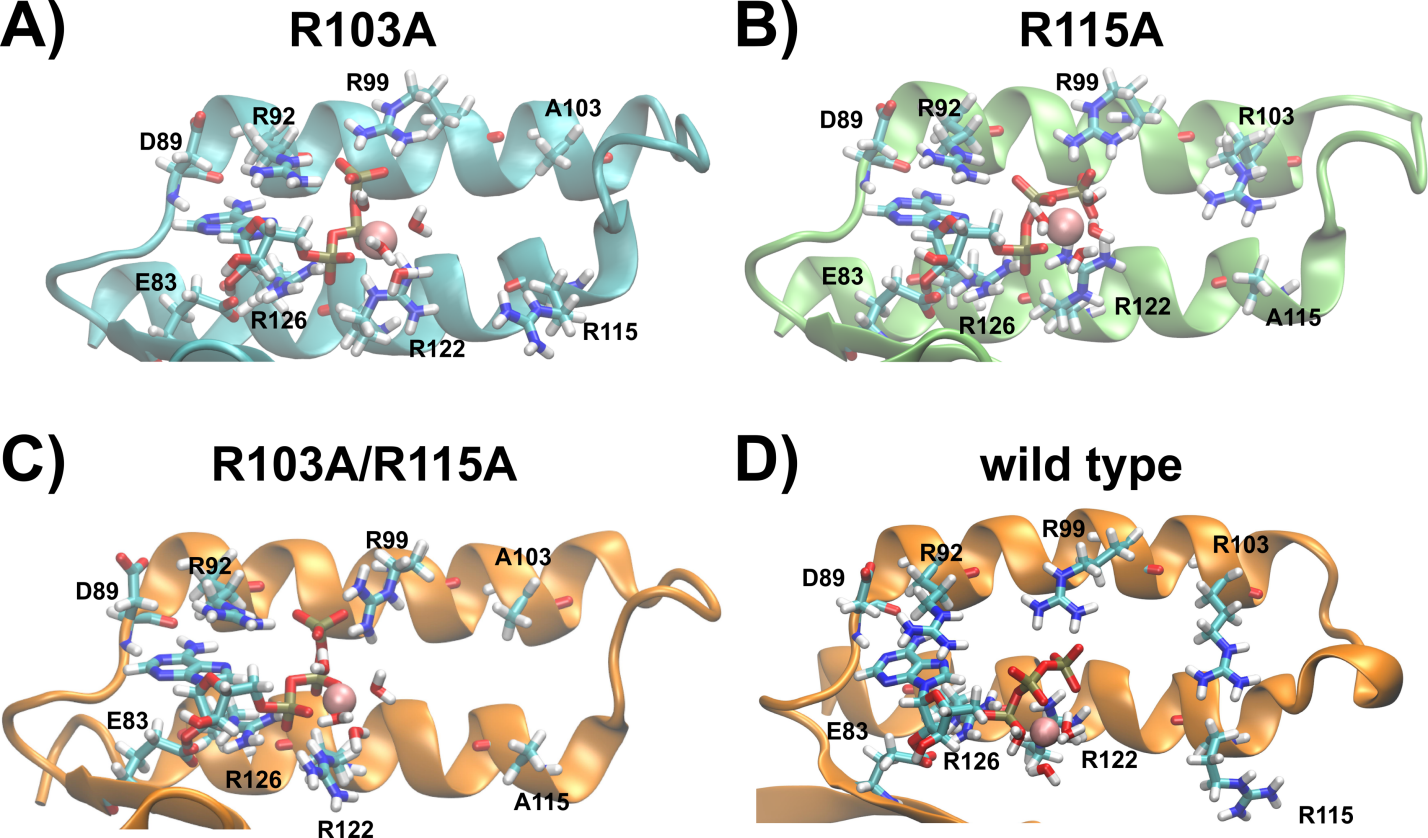
Predicted binding sites of the R103A and R115A mutants of the ε subunit from thermophilic *Bacillus* PS3. Zoom into the predicted binding site structures of the R103A, R115A R103A/R115A (Krah, Kato-Yamada & Takada, 2017) mutants and the wild type (Krah & Takada, 2016) of the ε subunit from thermophilic *Bacillus* PS3 are shown in (A), (B), (C) and (D), respectively. The protein is represented in cartoon format, with critical residues and bound Mg^2+^ATP shown in licorice format and labelled. (A) and (B) were rendered using VMD (Humphrey, Dalke & Schulten, 1996), while (C) and (D) were adapted from our previous work (Krah, Kato-Yamada & Takada, 2017). The predicted binding site structures of the R103A and R115A mutants when Mg^2+^ is bound to ATP:Oβ/O*γ* are shown in Fig. S6.

Taking all data derived from MD simulations into account, we predict the binding sites of the R103A and R115A mutants of the ε subunit from thermophilic *Bacillus* PS3 as follows: the nucleoside is stably coordinated and ATP:Oα interacts with R126:NHx in both mutants; the nucleoside-protein interactions are similar to the ones observed in the crystal structure. In the R103A mutant, a stable interaction of ATP:Oβ with R122:Nε and R122:NHx can be observed, while the R115A mutant shows an increased flexibility, but R122:NHx or R92:NHx coordinate ATP:Oβ. ATP:O*γ* is coordinated by R92:NHx and/or R99:NHx in both mutant proteins (Fig. S2). However, the R103A mutant additionally stabilizes the ligand via further interactions of R92:Nε with ATP:O*γ* (Fig. 3 (histogram) and S2 (timelines)). The R103A mutant binds ATP very similarly to the R103A/R115A mutant (Krah, Kato-Yamada & Takada, 2017) or the ε subunit from *C. thermarum* (Ferguson et al., 2016), while the R115A mutant binds in an analogous conformation as the wild type (Krah & Takada, 2016). The predicted structures of the ATP binding sites of both single mutants, the double mutant and the wild type from thermophilic *Bacillus* PS3 are shown in Fig. 5.

## Discussion

### ATP binding to the R103A and R115A mutants of the ε subunit from thermophilic *Bacillus* PS3

In light of previously obtained experimental (Kato, Yoshida & Kato-Yamada, 2007; Kato-Yamada, 2016) and theoretical data (Krah, Kato-Yamada & Takada, 2017) and results derived from this study, we propose that: (1) The single (R103A and R115A) mutants bind in the nM range, induced by an enhanced hydrogen bond network, as discussed previously for the R103A/R115A double mutant (Krah, Kato-Yamada & Takada, 2017). (2) Repulsive contacts (between positively charged binding site residues with other positively charged ATP binding protein residues or the Mg^2+^ ion coordinating ATP) further control the ligand binding affinity. (3) Whether the hydrogen bonding network of the 2nd α-helical domain (ranging from residues 112 to 133, Fig. 2, top orange) with the rest of the protein of the ε subunit is also stabilizing the ATP bound down-state has yet to be established, and awaits additional biochemical studies. It should be noted that the centre of mass distance between the two α-helices is 8.7 Å (wild type) <8.8 Å (R103A) <9.0 Å (R115A and R103A/R115A), as shown in Table S2; the centre of mass distance may also influence the binding affinity. The NMR structures of the apo state of the isolated ε subunit from *E. coli* (Wilkens et al., 1995; Wilkens & Capaldi, 1998) and *Thermosynechococcus elongates* BP-1 (Yagi et al., 2010) were resolved in the contracted down-state and thus indicate that the down-sate may be induced before ATP binds. However, in the ε*γ* sub-complex (Rodgers & Wilce, 2000) and the whole bacterial F_1_ domain (Cingolani & Duncan, 2011; Shirakihara et al., 2015), the ε subunit was found in a half-extended or fully extended state, respectively, and ATP sensors based on subunit ε show a conformational change upon ATP binding (Imamura et al., 2009; Yaginuma et al., 2014). Stabilization of the α-helical domain might be important, as the ATP bound down-state seems to require a certain length of the C-terminal domain, as SAXS experiments of the ε subunit from *Mycobacterium tuberculosis* identified the extended state of subunit ε in solution (Biukovic et al., 2013), which may be caused by the deletion of several residues in the C-terminal domain.

### A sequence analysis suggests substitutions at position 103 and 115 in the ε subunit from some organisms

**Figure 6:**
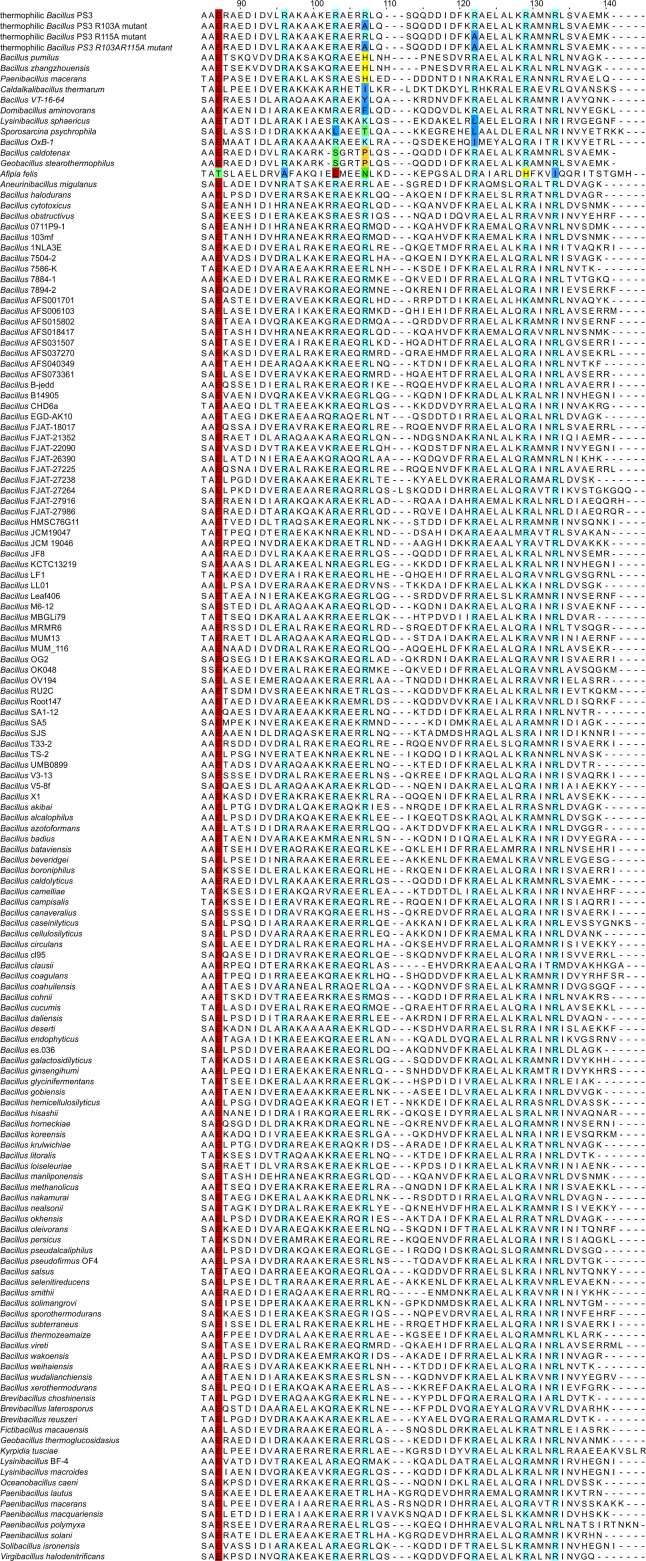
Excerpt of the sequence alignment of the ε subunits from different bacillic bacteria. Ligand binding residues E83 (E87), R92 (R96), R99 (R103), R122 (R129), R126 (R133) were found to be conserved. Substitutions in position 103 (107) and 115 (122) can be observed in the ε subunit from some organisms. Binding site residues, R103 and R115 from thermophilic *Bacillus* PS3 are highlighted; numbering in brackets denote the numbering in the alignment. The alignment was created with the program Jalview (Waterhouse et al., 2009). The whole alignment is shown in Fig. S7.

To explore if mutations of R103 and R115 are likely to occur naturally in other bacillic bacteria, we analysed the sequences of the ε subunit crystallized in the ATP bound state (thermophilic *Bacillus* PS3 (Yagi et al., 2007) and *C. thermarum* (Ferguson et al., 2016)), the studied mutants, and sequences deposited in the UniProt database (The UniProt Consortium, 2017); redundant sequences (threshold 95% identity) were removed from the sequence alignment. An excerpt of the sequence alignment, showing the critical sequence features of the binding sites, is shown in Fig. 6 (the whole alignment can be found in Fig. S7). We found that most of the sequences show the same binding site residues as thermophilic *Bacillus* PS3, also harbouring R103 and R115 (R107 and R122 in alignment, respectively). However, R103 and R115 seem to be highly conserved; R103 is, however, substituted in some more organisms, including *C. thermarum* (I103), which was crystallized recently (Ferguson et al., 2016). Thus the ε subunit from some bacillic organisms, carrying a mutation at positions 103 or 115 (sequence numbering *Bacillus* PS3), may bind ATP with remarkably increased affinity, similarly to the R103A/R115A double mutant. Thus, we also predict an increased ATP binding affinity for e.g., the ε subunits from *C. thermarum* and *Bacillus pumilus*. The ε subunit from these organisms harbour a leucine or a histidine at position 103 (instead of an arginine), respectively. However, it should be mentioned that these mutations may have different effects, as the histidine imidazole ring is titratable near neutral pH whilst leucine is a bulkier amino acid with respect to alanine and may lead to an increased number of hydrophobic interactions in comparison. Although the ε subunit from *Bacillus caldotenax* and *Geobacillus stearothermophilus* harbour a proline in position 103, we cannot yet confidently predict an increased (or decreased) binding affinity since one binding site residue (R99 in thermophilic *Bacillus* PS3) is mutated to serine, which may additionally influence its ligand binding affinity. As the ε subunit from *Lysinibacillus sphaericus* and *Bacillus* OxB-1 carry a hydrophobic substitution (L/I) at position 115 (*Bacillus* PS3 numbering), we also predict an increased ligand binding affinity for these proteins.

It should also be mentioned that the function of the ε subunit may have evolved due to environmental restraints. To function as an “emergency break”, the conformational transition from the ATPase inhibiting up-state to the non-inhibiting down-state of the ε subunit must sense the ATP concentration at roughly the same concentration under different cellular conditions, such as pH or temperature. Thus, the binding affinity at 65 °C, the physiological temperature of thermophilic *Bacillus* PS3 has been extrapolated to be 0.67 mM (Iino et al., 2005), comparable to the ATP binding affinity of the ε subunit of *Bacillus subtilis* (2.3 mM) at 25 °C (Kato-Yamada, 2005).

### Possible role of R103 and R115 in the wild type protein

Based on our previous results for the wild type protein (Krah & Takada, 2016), the R103A/R115A mutant (Krah, Kato-Yamada & Takada, 2017) and the findings in this study, we may speculate about the role of R103 and R115 in the wild-type protein. If ATP is stably bound to the wild type ε subunit from thermophilic *Bacillus* PS3, R103 and R115 may not coordinate ATP; however, R103 and/or R115 may bind to ATP during the ligand release process, causing unbinding of R122 (as observed in one run of the R115A mutant). Unbinding of R122 results in an unstable coordination of ATP bound to subunit ε, as a reduced affinity has been experimentally demonstrated for the R122A mutant of the ε subunit from thermophilic *Bacillus* PS3 (Kato, Yoshida & Kato-Yamada, 2007).

##  Supplemental Information

10.7717/peerj.5505/supp-1 Supplemental Information 1 Supporting Information

## Additional Information and Declarations

### Data Availability

The following information was supplied regarding data availability: The raw data are provided in the Supplemental File. Sequences were obtained from the publicly accessible UniProt database (www.uniprot.org; https://www.uniprot.org/uniprot/?query=atp+synthase+name%3Aepsilon+subunit+organism%3Abacillus&sort=score).
