# Supplementary material for: Single mutations in the ε subunit from thermophilic Bacillus PS3 generate a high binding affinity site for ATP
Source: PeerJ. 2018 Sep 5;6:e5505. doi: 10.7717/peerj.5505 (PMC6129141; doi:10.7717/peerj.5505)
Supplement: Supplemental Information 1 [file peerj-06-5505-s001.pdf]

Supporting Information

Single mutations in the ε subunit from thermophilic *Bacillus* PS3 generate a high binding affinity site for ATP

Alexander Krah[1,2*] and Peter J. Bond[3,4]

[1]School of Computational Sciences, Korea Institute for Advanced Study, 85 Hoegiro, Dongdaemun-gu, Seoul 02455, Republic of Korea

[2]Department of Biophysics, Graduate School of Science, Kyoto University, Kitashirakawa-Oiwakecho, Sakyo-ku, Kyoto, 606-8502, Japan

[3]Bioinformatics Institute, Agency for Science, Technology and Research (A*STAR), 30 Biopolis Str., #07-01 Matrix, Singapore 138671

[4] National University of Singapore, Department of Biological Sciences, 14 Science Drive 4, Singapore 117543

Table S1: Backbone RMSD of both helical domains in Å if ATP coordinates Mg^{2+} with the Oα and Oβ atoms.

	Wild type			R103A/R115A		
	**whole**	**1st helix**	**2nd helix**	**whole**	**1st helix**	**2nd helix**
**Run 1**	1.1 +/- 0.3	1.1 +/- 0.3	1.4 +/- 0.3	1.3 +/- 0.3	1.3 +/- 0.3	1.7 +/- 0.5
**Run 2**	1.6 +/- 0.4	1. 6 +/- 0.5	1.8 +/- 0.6	1.2 +/- 0.3	1.2 +/- 0.3	1.6 +/- 0.3
**Run 3**	1.2 +/- 0.4	1.2 +/- 0.3	1.4 +/- 0.4	1.4 +/- 0.4	1.4 +/- 0.5	1.0 +/- 0.3
**Average**	1.3 +/- 0.3	1.3 +/- 0.3	1.5 +/- 0.2	1.3 +/- 0.1	1.3 +/- 0.1	1.4 +/- 0.4

	R103A			R115A		
	**whole**	**1st helix**	**2nd helix**	**whole**	**1st helix**	**2nd helix**
**Run 1**	1.3 +/- 0.3	1.3 +/- 0.3	1.6 +/- 0.5	2.4 +/- 0.5	2.5 +/- 0.5	1.5 +/- 0.3
**Run 2**	1.2 +/- 0.3	1.2 +/- 0.3	1.4 +/- 0.3	1.4 +/- 0.3	1.4 +/- 0.3	1.5 +/- 0.5
**Run 3**	1.6 +/- 0.3	1.7 +/- 0.4	2.1 +/- 0.6	0.9 +/- 0.2	0.9 +/- 0.3	1.4 +/- 0.3
**Average**	1.4 +/- 0.2	1.4 +/- 0.3	1.7 +/- 0.4	1.6 +/- 0.7	1.6 +/- 0.8	1.5 +/- 0.06

Table S2: Center of mass (COM) distances of the α-helical domains in Å if ATP coordinates Mg^{2+} with the Oα and Oβ atoms. Average distance and standard deviation for this distance was derived by using runs 1 to 3.

**COM distance [Å]**	**Wild type**	**R103A**	**R115A**	**R103A/R115A**
**Run 1**	8.7 +/- 0.3	8.5 +/- 0.3	9.0 +/- 0.3	8.9 +/- 0.3
**Run 2**	8.6 +/- 0.4	9.0 +/- 0.2	9.2 +/- 0.3	9.0 +/- 0.3
**Run 3**	8.8 +/- 0.3	8.8 +/- 0.4	8.9 +/- 0.3	9.0 +/- 0.3
**Average**	8.7 +/- 0.1	8.8 +/- 0.3	9.0 +/- 0.2	9.0 +/- 0.06

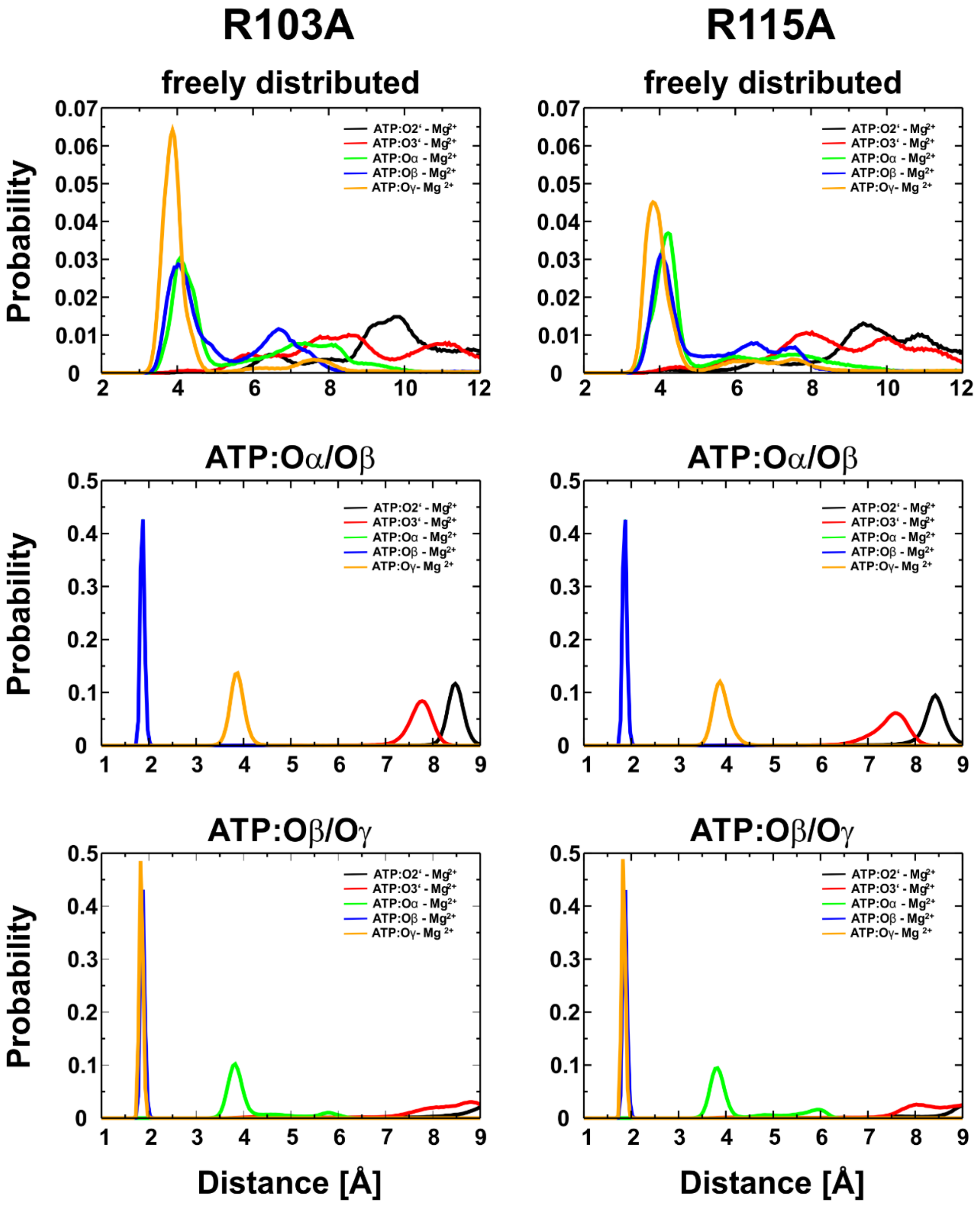

Figure S1: Distance of the Mg^{2+} to ATP oxygen atoms, when ATP is bound to the ε subunit from thermophilic *Bacillus* PS3 mutants (R103A and R115A). The top graphs show how Mg^{2+} binds to these oxygen atoms, when the ion is freely distributed. The middle and bottom graphs show the distance distributions, when Mg^{2+} is bound to ATP:Oα/Oβ or ATP:Oβ/Oγ, respectively. It should be noted that two histograms (Mg^{2+} - ATP:Oα and Mg^{2+} - ATP:Oβ) are overlaid if Mg^{2+} is coordinated to ATP:Oα/Oβ (middle row). The results for the R103A and R115A mutants are shown in the left and right coloums, respectively.

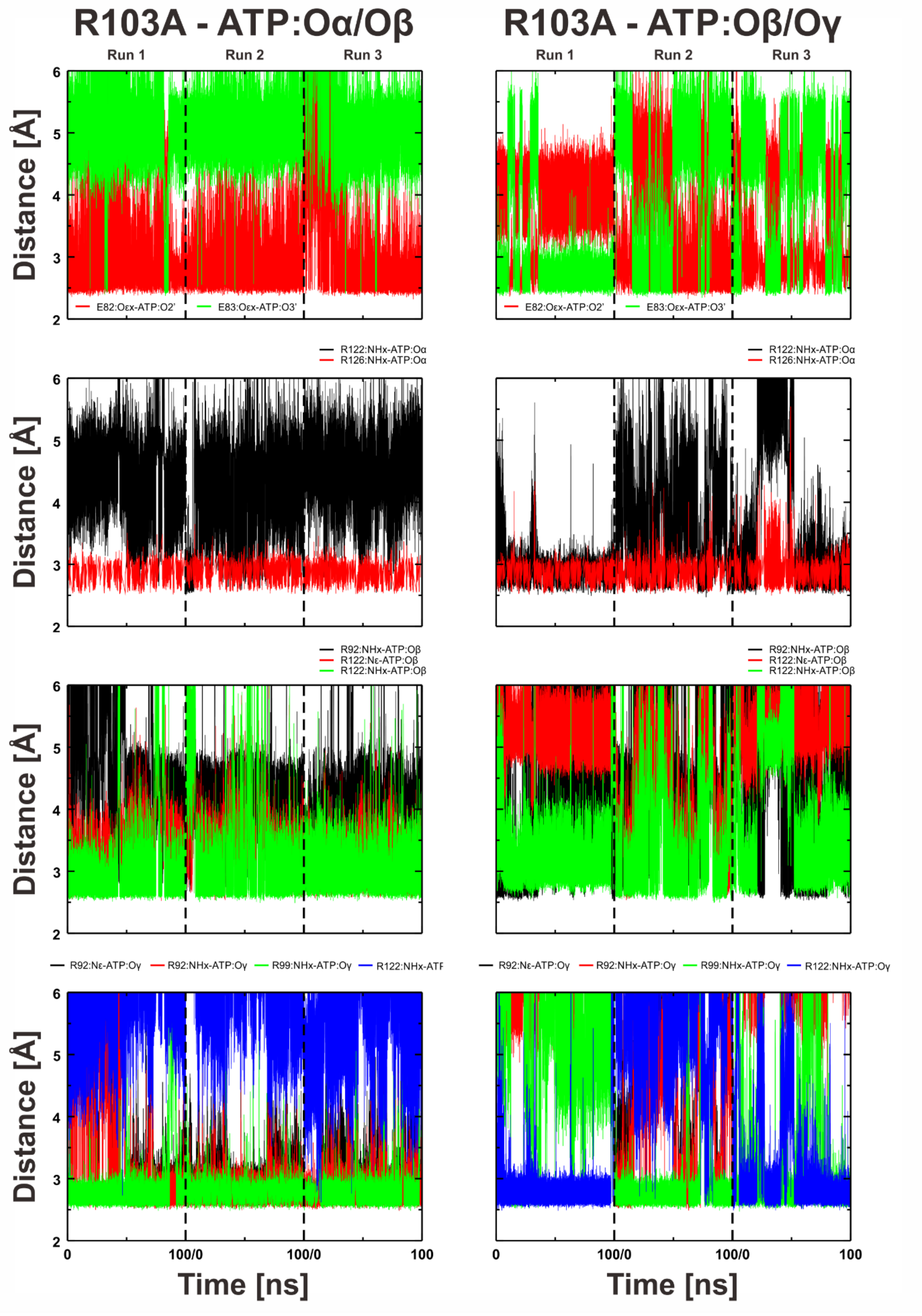

R103A - ATP:Oα/Oβ
R103A - ATP:Oβ/Oγ
Run 1
Run 2
Run 3
Distance [Å]
E82:Oεx-ATP:O2'
E83:Oεx-ATP:O3'
R122:NHx-ATP:Oα
R126:NHx-ATP:Oα
R92:NHx-ATP:Oβ
R122:Nε-ATP:Oβ
R122:NHx-ATP:Oβ
R92:Nε-ATP:Oγ
R92:NHx-ATP:Oγ
R99:NHx-ATP:Oγ
R122:NHx-ATP:Oγ
0
100/0
100
Time [ns]

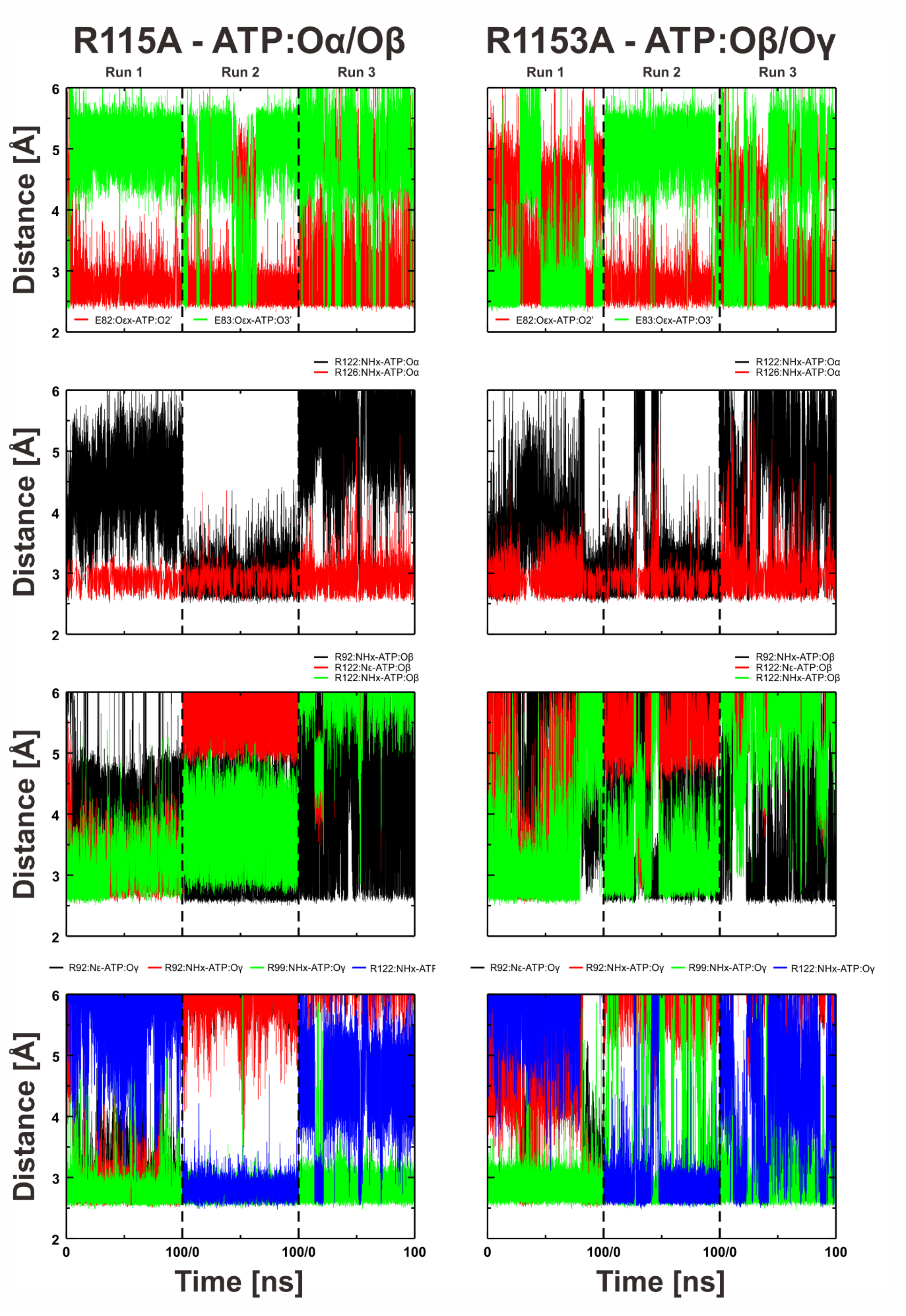
R115A - ATP:Oα/Oβ
R1153A - ATP:Oβ/Oγ
Run 1
Run 2
Run 3
Distance [Å]
E82:Oεx-ATP:O2'
E83:Oεx-ATP:O3'
R122:NHx-ATP:Oα
R126:NHx-ATP:Oα
R92:NHx-ATP:Oβ
R122:Nε-ATP:Oβ
R122:NHx-ATP:Oβ
R92:Nε-ATP:Oγ
R92:NHx-ATP:Oγ
R99:NHx-ATP:Oγ
R122:NHx-ATP:Oγ
0
100/0
100
Time [ns]

Figure S2: Timelines of relevant interactions of the R103A and R115A single mutants of the ε subunit from thermophilic *Baillus* PS3. For each mutant the state in which Mg^{2+} is coordinated by ATP:Oα/Oβ and ATP:Oβ/Oγ is shown on the left and right , respectively

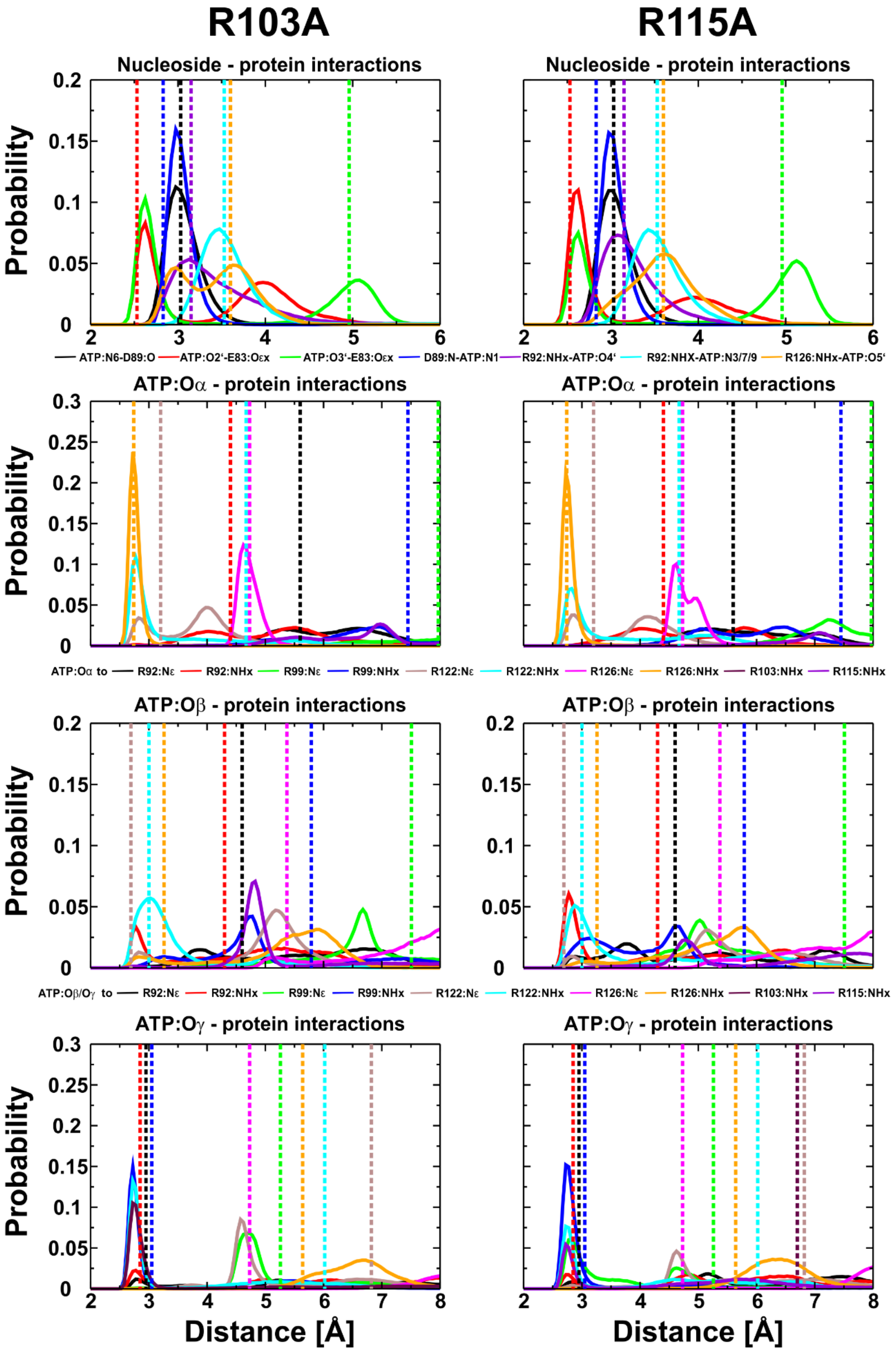

R103A
R115A
Nucleoside - protein interactions
Probability
ATP:N6-D89:O
ATP:O2'-E83:Oεx
ATP:O3'-E83:Oεx
D89:N-ATP:N1
R92:NHx-ATP:O4'
R92:NHX-ATP:N3/7/9
R126:NHx-ATP:O5'
ATP:Oα - protein interactions
ATP:Oα to
R92:Nε
R92:NHx
R99:Nε
R99:NHx
R122:Nε
R122:NHx
R126:Nε
R126:NHx
R103:NHx
R115:NHx
ATP:Oβ - protein interactions
ATP:Oβ/Oγ to
ATP:Oγ - protein interactions
Distance [Å]

Figure S3: Interactions of the ε subunit R103A (left) and R115A (right) mutants from thermophilic *Bacillus* PS3 with ATP during the simulations when Mg^{2+} is coordinated by ATP:Oβ/Oγ. Dotted lines correspond to the distances observed in the crystal structure of the wild type protein.

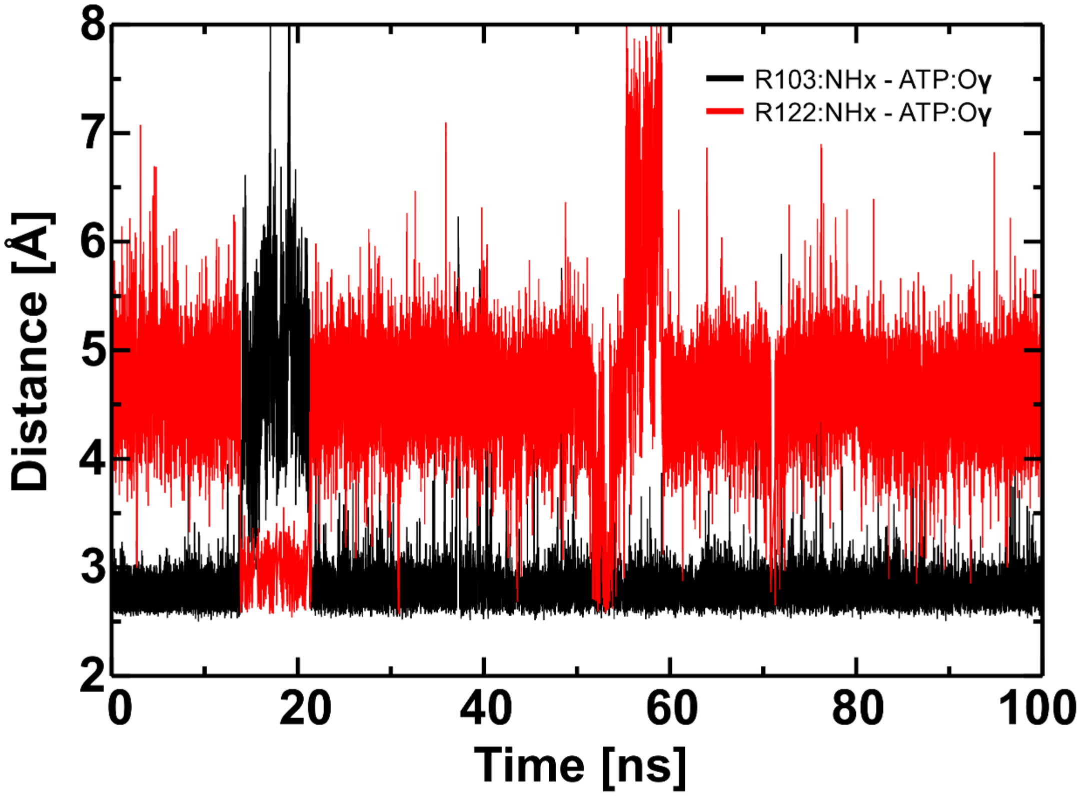

Figure S4: Interactions of R103:NHx (black) and R122:NHx (red) with ATP:Oγ, respectively of the excluded run of the R115A mutant. In this run, it can be observed that R103 binding to the ligand leads to a release of R122 interactions with ATP.

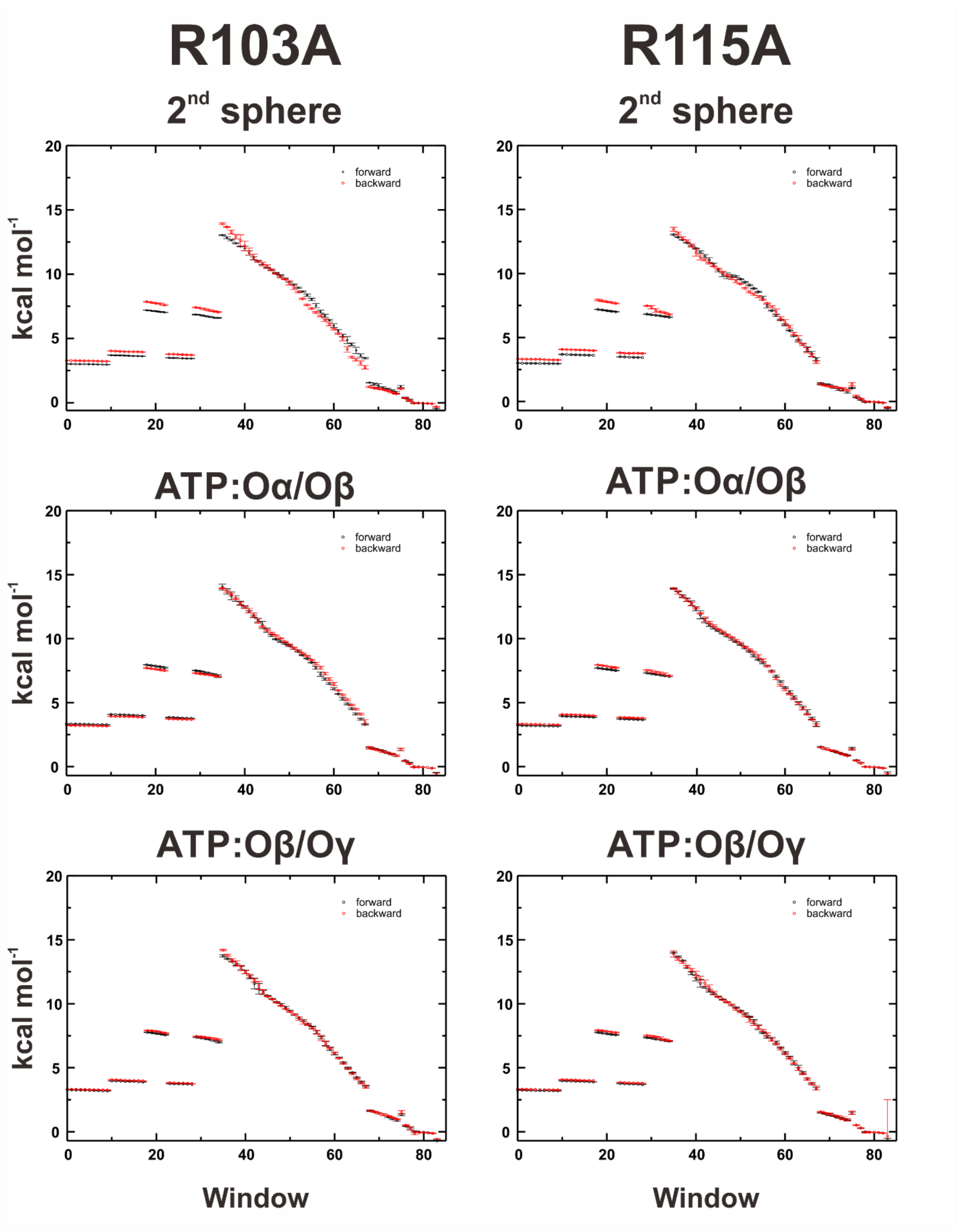

Figure S5: Energetic contributions of the single windows in both mutants for all TI calculations.

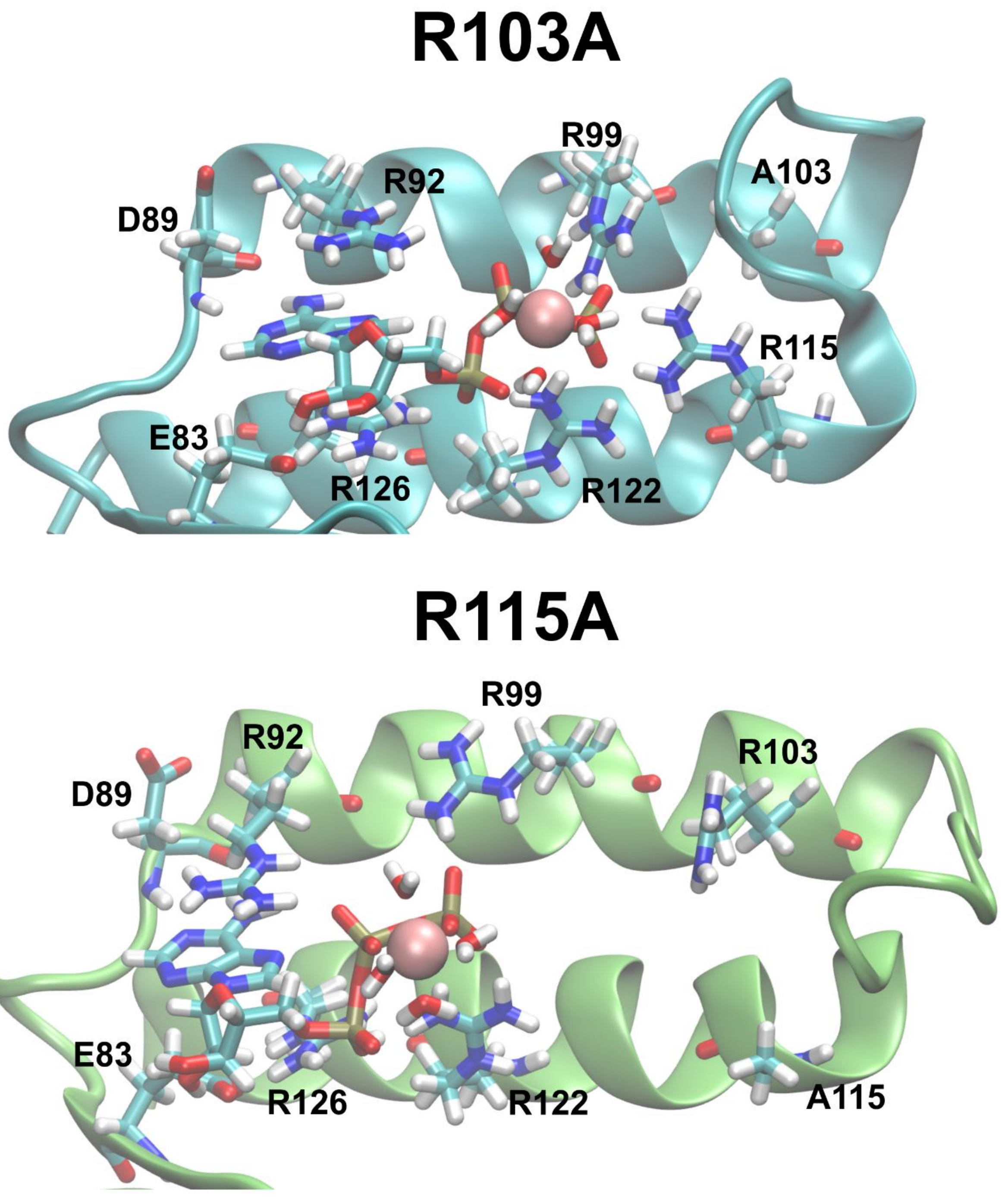

Figure S6: Predicted binding sites of the R103A and R115A mutants of the ε subunit from thermophilic *Bacillus* PS3, in the hypothetical case if the ion is bound to ATP:Oβ/Oγ.

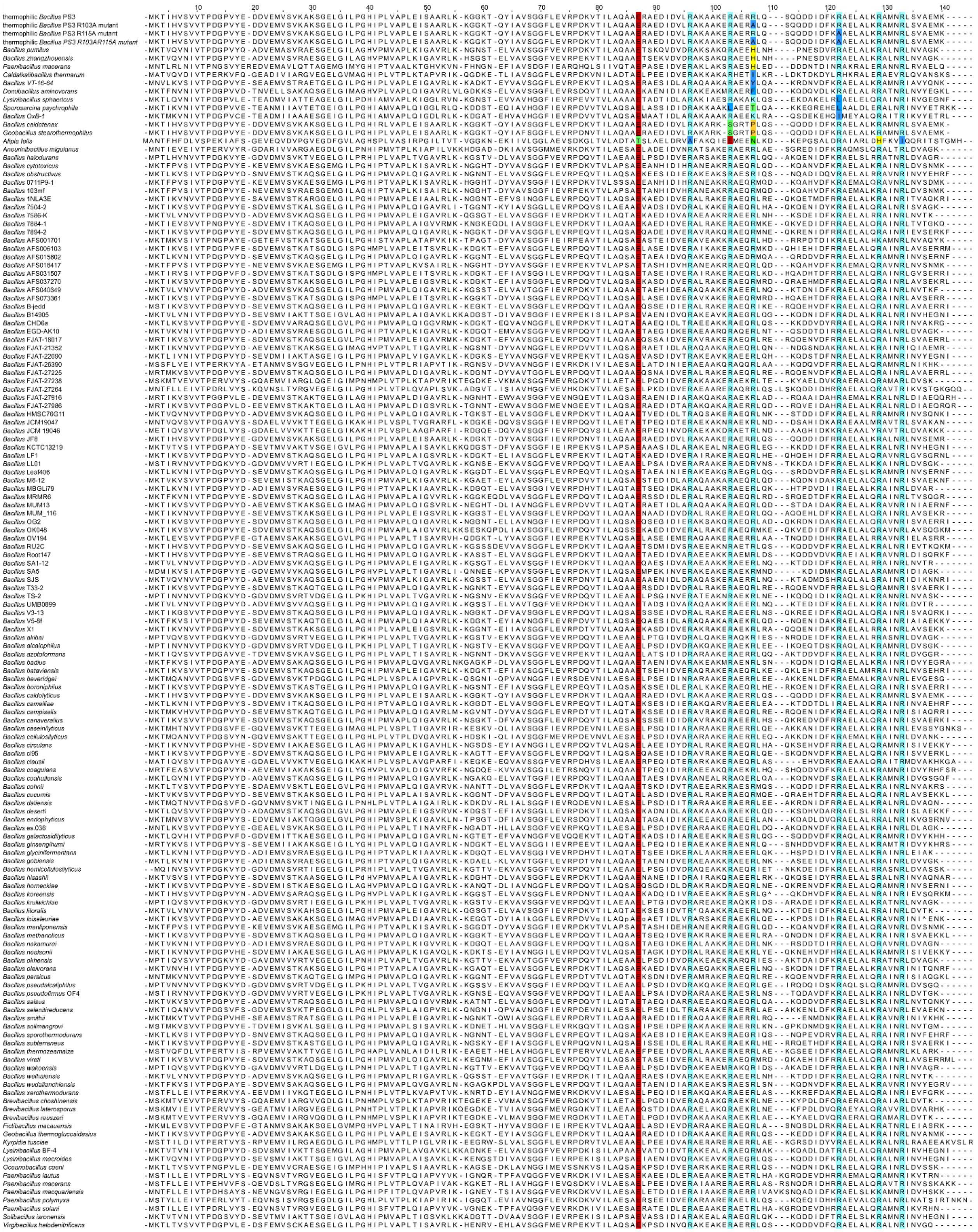

Figure S7: Sequence alignment of the whole sequence of ε subunits from different bacillic bacteria.
